# Weight Perception Measured by Verbal Descriptions and Visual Descriptions: Which Measurement Correlates with Weight Loss Intentions among Female Nursing Students?

**DOI:** 10.3390/ijerph18105200

**Published:** 2021-05-13

**Authors:** Ruxing Wu, Bingqian Zhu, Rongfeng Chen, Liqun Chen, Runan Chen, Daqiao Zhu

**Affiliations:** 1 Shanghai Jiao Tong University School of Nursing, Shanghai 200025, China; wu_ruxing@163.com (R.W.); zhubq@shsmu.edu.cn (B.Z.); 2 School of Nursing and Health Management, Shanghai University of Medicine & Health Sciences, Shanghai 201318, China; chenrf@sumhs.edu.cn; 3 School of Nursing, Fudan University, Shanghai 200032, China; liqunchen@fudan.edu.cn; 4 School of Nursing, The Second Military Medical University, Shanghai 200433, China; crnsweet@163.com

**Keywords:** weight perception, overestimation, female, college students, nursing, weight loss intention

## Abstract

Background: Young females tend to overestimate their weight status, which might induce unhealthy weight loss intentions and behaviours. This study aimed to examine weight perception measured by visual and verbal descriptions and its correlation with weight loss intentions among female nursing students. Methods: A cross-sectional survey was conducted among 600 female nursing students from four medical colleges in Shanghai, China. The participants rated perceptions of their weight by selecting a silhouette from the female Photographic Figure Rating Scale (PFRS) and one of the following verbal descriptions: “very underweight”, “slightly underweight”, “normal”, “overweight” or “obese”. Weight loss intentions were measured using the question “How often do you want to lose weight?”. Body mass index (BMI) was calculated from self-reported height and weight. Data were analysed using univariate and ordinal logistic regression analyses. Results: The accuracy of weight perceptions measured by verbal descriptions and visual descriptions was 44.50% and 55%, respectively. In females with underweight BMI (*n* = 135), 88.15% and 49.63% accurately classified their weight using visual descriptions and verbal descriptions, respectively. These females were more likely to overestimate (53.83% vs. 14.50%) and less likely to underestimate (1.67% vs. 30.50%) their weight when using verbal descriptions than when using visual descriptions. For verbal descriptions, weight overestimation was associated with weight loss intentions (odds ratio, 1.80; 95% confidence interval, 1.25–2.60). However, for visual descriptions, the two variables were not associated. Conclusions: A mismatch occurred between weight perceptions measured by the two methods and BMI status among female nursing students. Compared with verbal descriptions, visual descriptions had higher weight perception accuracy. However, weight overestimation measured by verbal descriptions was more likely to be associated with stronger intentions to lose weight than that of visual descriptions. These findings suggest that methodological discrepancies should be taken into account when measuring weight perception in future studies.

## 1. Introduction

Weight perception refers to an individual’s perception of their body size, shape, and weight status related to body weight [1,2]. Weight misperception occurs when there is a discrepancy between one’s body mass index (BMI) status and perceived weight status. It can be further classified into weight overestimation (perceived weight status > objectively measured/self-reported BMI status) and weight underestimation (perceived weight status < objectively measured/self-reported BMI status) [3].

A growing number of studies have consistently shown that individuals’ weight perceptions are inaccurate [4,5,6,7,8]. According to the Health Survey for England, among overweight and obese individuals, the proportion of people who underestimated their weight increased from 1997 to 2015 (males: 37% to 40%; females: 17% to 19%) [4]. Based on the National Health and Nutrition Examination Survey of the United States (1999–2016) [5], an increase in the proportion of people who perceived themselves as underweight or about the right weight increased among those with a BMI of at least 25 kg/m2 (29.5% to 32.8%). In addition, a survey of eight cities in China revealed that the prevalence of weight misperception was 21.3% [6].

Both weight overestimation and underestimation might provide misleading population health estimations, resulting in negative impacts on personal attitudes or behaviours related to weight, and even leading to physical and psychological consequences [9,10]. People with obesity who underestimated their body weight were found to be less sensitive to weight-related health risks and not actively engage in weight loss behaviours [11,12]. In contrast, people with normal weight who overestimated their weight status adopted extreme efforts to lose weight, such as restrictive dieting, excessive exercise or laxative use [9], which increased risks for body dissatisfaction and eating disorders, especially among young females who are usually thin [10,13].

Notably, weight perception measurements adopted in those studies were different. Weight perception could be measured by two types of measures that differ in the type of prompt [1]. First, it can be measured using verbal descriptions, including the Body Image Distortion Questionnaire (BIDQ) [14] and the Youth Risk Behavior Surveillance System (YRBSS) [15]. This type of method measures perceived weight by asking participants to describe their current weight on a rating scale. Second, weight perception can also be measured using visual descriptions, such as the Stunkard Figure Rating Scale (FRS) [16] and the female Photographic Figure Rating Scale (PFRS) [17]. This type of method presents a visual prompt in the form of images of human figures and measures perceived weight by asking participants to select from a range of figures/silhouettes. Using different methods in different studies likely affects the results of weight perception accuracy. Tanenbaum et al. (2015) [13] analysed data from a longitudinal study conducted in China and reported that 42.2% (317/751) of the female college students who were underweight or had normal weight perceived themselves as overweight/obese as measured by verbal descriptions. In contrast, in another study conducted in six Chinese universities (*n* = 1005) [18], the average self-reported BMI was 20.09 kg/m^2^ (normal weight) but the perceived weight status measured by visual descriptions was underweight. This finding indicates that female college students may underestimate their weight. The large discrepancies between the two studies may be due to the methods used to measure weight perception. Little attention has been given to whether and to what extent different methods for measuring weight perception may influence weight perception accuracy. Examining agreement between the two methods with the same sample is necessary and may help explain inconsistent results.

Existing studies have focused mainly on weight underestimation and less on weight overestimation because of the increasing prevalence of overweight and obesity worldwide in recent decades [4,11,19]. However, weight overestimation is common in some groups, especially in young females [13,20,21,22]. Therefore, examining weight overestimation is particularly relevant among female college students. Recently, a systematic review provided strong evidence that perceived overweight was associated with a higher likelihood of intending or attempting to lose weight, especially in those with normal weight (that is, weight overestimation was associated with weight loss intentions/attempts in this group) [23]. However, in the above-mentioned systematic review, weight perception in the included studies was measured by verbal descriptions, and the relationship between weight misperception measured by visual descriptions; weight loss intentions/attempts were not examined. More importantly, prior studies [13,22,23,24] only controlled for sociodemographic (e.g., age, gender or family income) and health-related factors (e.g., smoking, drinking or stress level), but ignored potential confounders, including peers’ attitudes and comparisons, mass media, and social stereotypes. Those constructs have been reported to be associated with individuals’ weight perceptions and weight loss intentions/behaviours [25,26,27,28]. Collectively, it is necessary to examine whether weight misperception measured by both verbal and visual descriptions were related to weight loss intentions or behaviours, while adjusting for potential confounders.

Building on previous evidence, this study aimed to (1) investigate whether weight perceptions measured by visual and verbal descriptions were related to BMI status and their agreement with BMI status, and (2) compare the extent to which weight perception measured by visual descriptions and verbal descriptions could be related to weight loss intentions. Findings from this study will provide further evidence for the selection of weight perception measurement when examining its association with weight loss intentions.

## 2. Materials and Methods

### 2.1. Study Population

A cross-sectional survey was conducted anonymously between March and April 2019. Online data were collected through a professional questionnaire survey platform (Wenjuanxing, www.sojump.com (accessed on 30 April 2021)) from March to April in 2019. The questionnaire link was disseminated via WeChat APP. The participants were selected from four medical colleges in Shanghai, China, using convenience sampling. The inclusion criteria were as follows: (1) full-time female nursing students and (2) willingness to participate in this study. The exclusion criteria were as follows: (1) suspension of schooling during the investigation, (2) pregnancy during the investigation, (3) severe mental/neurological diseases, and (4) acute/chronic organic diseases. The participants completed informed consent prior to data collection.

A total of 725 questionnaires were collected, 600 of which were valid and 125 were invalid, resulting in an 82.76% effectiveness rating. The criteria for judging invalid questionnaires were: (1) obvious regular answers, that is, all items in a scale had the same answer, and (2) illogical responses. Our sample size was comparable to a previous study among Hispanic college females which found a significant relationship between weight perception and weight loss intentions (*n* = 467) [24].

### 2.2. Measures

#### 2.2.1. Sociodemographic and Health-Related Factors

We collected the following self-reported sociodemographic information: age, height, weight, nationality, religion, smoking, drinking, stress level, residence (rural/urban), living area (south/central/north), parents’ level of education, and family income.

#### 2.2.2. BMI Status

Although BMI status can be measured using objective measurements, self-reported BMI status has been commonly used in large-scale studies [5,9,20]. We thus obtained self-reported weight and height to calculate BMI using the following formula: BMI = weight (kg)/height(m)^2^. We used the Guidelines for Prevention and Control of Overweight and Obesity in Chinese Adults to define the following categories: underweight (BMI < 18.5 kg/m^2^), normal weight (18.5 kg/m^2^ ≤ BMI ≤ 23.9 kg/m^2^), overweight (24.0 kg/m^2^ ≤ BMI ≤ 27.9 kg/m^2^), and obese (BMI ≥ 28.0 kg/m^2^) [29].

#### 2.2.3. Weight Perception

**Figure 1:**
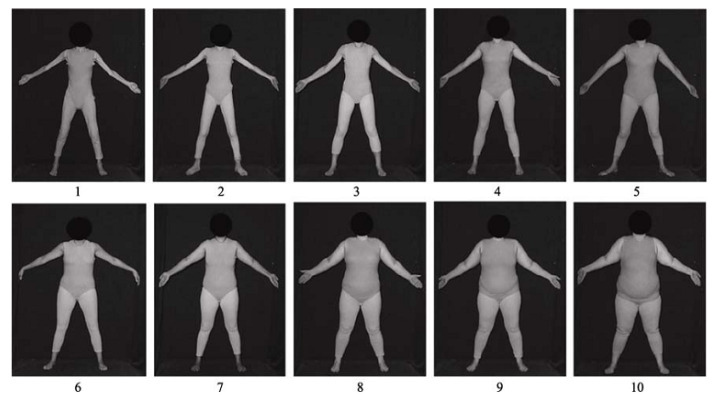
The female Photographic Figure Rating Scale (PFRS).

Two methods were used to measure weight perception. First, verbal descriptions were used by asking participants, “How would you describe your own weight status?” This question was adopted from the Youth Risk Behavior Surveillance System (YRBSS) [15]. The participants chose the category that they thought best described themselves among the provided weight categories: very underweight, slightly underweight, normal weight, overweight, and obese. Second, visual descriptions were used by asking participants to select a silhouette from the female PFRS (Figure 1) [17]. This scale includes 10 front-view images of real females with BMI ranging from the lowest BMI (“1”) to the highest BMI (“10”); all depicted females wear grey tights, have their heads obscured and stand in a set posing at a standard distance so that the influence of other factors such as facial appearance and race is eliminated [18]. Table 1 provides each image’s BMI. The participants were asked to select the silhouette that most closely resembled themselves. The corresponding BMI was recorded. The numbers between adjacent integers (such as 2.5 or 3.6) could also be selected. In addition, the participants also selected the silhouette that represented their ideal body shape.

We calculated a new variable weight misperception, which refers to the difference between the participant’s BMI status and perceived weight status. Two types of weight misperception were obtained: one was the difference between BMI status and perceived weight status measured by verbal descriptions, and the other one was the difference between BMI status and perceived weight status measured by visual descriptions. Each weight misperception was divided into three categories: (1) accurate [perceived weight status = self-reported BMI status], (2) underestimated [perceived weight status < self-reported BMI status], and (3) overestimated [perceived weight status > self-reported BMI status].

#### 2.2.4. Weight Loss Intention

We also measured weight loss intention frequency by asking participants “How often do you want to lose weight?”. They selected the most suitable option from five choices: always, often, sometimes, seldom, and never.

#### 2.2.5. Other Variables

Stereotypes about obesity: the Chinese version of the Attitudes Toward Obese Persons (ATOP) scale with a 12-item 6-point Likert scale was used to investigate individuals’ stereotyped views about obesity [30]. The response options ranged from −3 (“strongly disagree”) to +3 (“strongly agree”). The total possible score of this ATOP version is 36 plus the sum of the 12-item scores to eliminate negative scores (the total score of the Chinese version ranges from 0–72). Higher scores indicate more positive attitudes, while lower scores indicate more negative attitudes towards obese persons. In this study, the internal consistency reliability evaluated by Cronbach’s alpha was 0.703.

Peers’ attitudes and comparisons: a subscale of the Tripartite Influence Scale developed by Keery et al. (2004) [31] was used to evaluate the influence of peers’ attitudes towards weight, weight loss, and appearance on individuals. It consists of 12 items with a 5-point Likert-type response scale ranging from “never” (1) to “always” (5). Higher scores indicate greater negative influences from peers. In this study, the internal consistency reliability evaluated by Cronbach’s alpha was 0.898. Peers’ comparison was measured by asking participants the following question: “Among slightly fat, moderate, and slightly thin, which describes how you think of yourself compared to your peers?”

Media influence on ideal body shape: an item developed by Field et al. (1999) [28] was used to assess the media influence on ideal body shape. The participants were asked, “Do you think that pictures of females in magazines influence what you think is the perfect body shape?”. They selected from “high”, “moderate” or “infrequent”.

### 2.3. Data Analysis

Statistical analyses were performed using IBM SPSS Statistics for Windows (Version 24.0, IBM Corporation, Armonk, NY, USA). There were no missing data. The normality of data distribution was checked. The prevalence of weight misperception, measured by the two methods and demographic characteristics among female nursing students, was analysed using descriptive analyses. Continuous variables were presented as mean and standard deviation (SD). Categorical variables were presented as frequency and percentage. Agreement between BMI status and weight perception was assessed using the kappa statistic. One-way analysis of variance (ANOVA) and the chi-square test were used to analyse the correlation between the above-mentioned factors and weight loss intentions.

In this study, the weight loss intention was an ordered five-category dependent variable. Thus, ordinal logistic regression was conducted to retain more data, with weight misperception as the independent variable (accurately perceived weight status as the reference group). We also controlled for possible confounders, including demographic characteristics, stereotypes about obesity, peers’ attitudes and comparisons, and media influence on ideal body shape. One aim of this study was to compare the extent to which weight perception measured by visual descriptions and verbal descriptions could be related to weight loss intentions. It requires that each subgroup (i.e., underestimation, accurate estimation, and overestimation) measured by both methods should have enough sample for analysis. Only 10 participants (1.67%) underestimated their weight by verbal descriptions. This subgroup was not adequate for logistic regression. We thus excluded the underestimation subgroup measured by visual descriptions as well (*n* = 183). The final ordinal logistic analysis was limited to the accurate estimation and overestimation subgroups. After the final model was obtained, a score test for the proportional odds assumption, the likelihood ratio test, and collinearity diagnostics were performed. All statistical analyses were carried out with a 95% confidence interval (CI). Statistical significance was set at *p*-value < 0.05 (two-tailed).

## 3. Results

A total of 600 female nursing students were included in our study. No significant differences in height, weight, BMI status, religion, nationality, living area, residence, parents’ level of education, family income, or weight loss intentions between the included and excluded groups were found. However, participants not included in the analysis [mean (SD): 19.63 (1.32)] tended to be younger than those included in the analysis [mean (SD): 20.00 (1.33)] (*p* = 0.006). Table 2 details the sample’s demographic characteristics. The participants’ ages were between 16 and 28 years, with an average of 20 years (SD = 1.33); BMIs were between 14.76 and 33.20 kg/m^2^, with an average of 20.67 kg/m^2^ (SD = 2.82), and divided into four categories: underweight (*n* = 135 [22.50%]), normal weight (*n* = 394 [65.67%]), overweight (*n* = 60 [10.00%]), and obese (*n* = 11 [1.83%]).

Table 3 presents the comparison between perceived weight status and BMI status. Using verbal descriptions, 55.50% of female nursing students did not accurately perceive their weight status, while 45.00% did not accurately perceive their weight status using visual descriptions. The consistency between BMI status and perceived weight status was poor (the kappa value of verbal descriptions was 0.195 and that of visual descriptions was 0.304). Less than half of the participants (49.63%) accurately identified themselves as underweight when using verbal descriptions, whereas 88.15% accurately identified themselves as underweight when using visual descriptions. Table 4 compares weight misperception between the two methods. Overall, using verbal descriptions, very few participants underestimated their weight (1.67%), while up to 53.83% overestimated their weight. Using visual descriptions, the prevalence of weight underestimation was 30.50%, and weight overestimation accounted for only 14.50% of the total. In addition, among participants who overestimated their weight using verbal descriptions, 56.97% accurately perceived their weight status using visual descriptions.

Table 5 shows the ordinal logistic regression analyses’ results for the association between weight misperception (overestimated vs. accurate) measured by the two methods and weight loss intentions. In this study, weight loss intention was the dependent variable (1 = never, 2 = seldom, 3 = sometimes, 4 = often, 5 = always) and weight misperception was the independent variable, with accurately perceived weight status as the reference. Variables with statistical significance in the bivariate analyses were also included in the ordinal logistic regression model.

(1) Regarding weight overestimation based on verbal descriptions, the univariate analyses showed that weight misperception [χ2 = 57.21, *p* < 0.001], BMI status [χ2 = 99.70, *p* < 0.001], self-perceived weight level among peers [χ2 = 128.64, *p* < 0.001], father’s level of education [χ2 = 25.40, *p* = 0.013], media influence on ideal body shape [χ2 = 273.66, *p* < 0.001], stress level [χ2 = 51.56, *p* < 0.001], family monthly income [χ2 = 17.99, *p* = 0.021], ATOP score [F = 11.19, *p* < 0.001], and peers’ attitudes [F = 21.12, *p* < 0.001] were statistically significant. The ordinal logistic regression analysis showed that weight overestimation was the independent risk factor for weight loss intentions (OR = 1.80, 95% CI: 1.25–2.60). That is, after controlling for BMI status, peers’ attitudes, and other factors, female nursing students who overestimated their weight had weight loss intentions more frequently than those who accurately perceived their weight status.

(2) Regarding weight overestimation based on visual descriptions, the bivariate analyses showed that weight misperception [χ2 = 63.23, *p* < 0.001], BMI status [χ2 = 88.78, *p* < 0.001], self-perceived weight level among peers [χ2 = 119.04, *p* < 0.001], father’s level of education [χ2 = 25.86, *p* = 0.011], mother’s level of education [χ2 = 30.58, *p* = 0.002], media influence on ideal body shape [χ2 = 183.90, *p* < 0.001], stress level [χ2 = 45.38, *p* < 0.001], family monthly income [χ2 = 25.43, *p* = 0.001]), ATOP score [F = 6.85, *p* < 0.001], and peers’ attitudes [F = 19.32, *p* < 0.001] were statistically significant. The ordinal logistic regression analysis showed that weight misperception was not related to weight loss intentions (OR = 1.14, 95% CI: 0.70–1.86).

## 4. Discussion

In this study, we found that weight perception accuracy measured by verbal descriptions and weight perception accuracy measured by visual descriptions were low and different; the former overestimated and the latter underestimated each participant’s BMI status. We also found that weight overestimation based on verbal descriptions as opposed to visual descriptions, was associated with higher weight loss intention frequency among female nursing students. These novel findings have important implications for selecting a suitable weight perception measurement in further studies.

Female nursing students had low rates of accurate weight perception regardless of which measurement method was used and weight perception accuracy measured by visual descriptions was higher than that of verbal descriptions (55.00% vs. 44.50%, *p* < 0.001). Our result is similar to those of previous studies [13,18], which showed that of 902 female college students in China [13], only 53.88% had accurate weight perceptions measured by verbal descriptions. In our study, female nursing students were more likely to overestimate their weight when verbal descriptions were used, while female nursing students using visual descriptions were more likely to underestimate their weight. Female college students’ ideal body shape is mostly thin, which is influenced by social norms and expectations (in this study, up to 89.67% of female nursing students chose underweight as their ideal body shape). Participants who use verbal descriptions may consider ideal body shapes as the reference value of normal weight, thereby overestimating their own weight, which is supported by the Visual Normalization Theory [2]. This theory states that individuals often perceive their weight status in the context of visual body-weight norms. Norms are shaped by the body size a person is dominantly exposed to in their environment. As society and mass media increasingly promote the perfect figure and people become more frequently exposed to an underweight environment, their visual body-weight norms are recalibrated [2]. Therefore, when using verbal descriptions, female nursing students would normalize the perfect figure (i.e., underweight), use it as a reference, and eventually overestimate their weight. However, for visual descriptions, Zhou et al. (2014) [18] found that female college students tended to overestimate the BMIs of corresponding images. This finding may help explain why nearly half of the female nursing students with normal weight in this study underestimated their weight. It was not that these students thought they were underweight, but rather that they overestimated the weight status of the silhouette in the female PFRS. A study using visual descriptions among university students in Morocco found that 2.27% (3/132) of female college students overestimated their weight and that 39.39% (52/132) underestimated their weight [32]. Although the prevalence of weight overestimation was much lower than that found in this study (87/600 [14.5%]), the prevalence of weight underestimation was similar (183/600 [30.50%]). Thus, when visual descriptions were used to measure weight perception, an individual was likely to underestimate their weight instead of overestimate it.

In this study, weight misperception (overestimated vs. accurate) measured by verbal descriptions as opposed to visual descriptions, was independently correlated with female nursing students’ weight loss intentions. For verbal descriptions, we found that participants who overestimated their weight reported more frequent weight loss intentions than those who did not overestimate their weight. A systematic review by Haynes et al. (2018) [23] suggested that individuals who perceived their weight status as overweight when using verbal descriptions were more likely to report weight loss attempts and eating disorders. Nissen and Holm (2015) [8] also found that in many studies, the difference between perceived weight status measured by verbal descriptions and the actual measured weight status led to individuals’ desire for weight loss. One explanation could be that when using verbal descriptions, female college students believe that they do not meet the ideal body shape standard, and they, therefore, are more likely to have strong weight loss intentions. In our study, for 86.61% of the participants, perceived weight status measured by verbal descriptions was higher than the ideal body shape. This finding indicates that this measurement of weight perception can objectively reflect individuals’ actual weight loss intentions. However, although weight misperception measured by visual descriptions was statistically correlated with weight loss intentions in the univariate analysis, the correlation disappeared after multiple covariates were controlled in the logistic regression model. This finding showed that although weight misperception measured by visual descriptions was related to weight loss intentions, it was not the main factor; it might have interactions with other factors. Future research is warranted to confirm that weight perception measured by verbal descriptions rather than visual descriptions would predict weight loss intentions and behaviours.

## 5. Limitations

Our findings add to current knowledge concerning agreement between the two weight perception measures and the relationship between weight misperception and weight loss intentions. To the best of our knowledge, this study was among the first to demonstrate and compare the relationship between weight misperception measured by two methods and weight loss intentions. However, there are limitations to our study. First, the study used a cross-sectional design, limiting the causal inference. Second, our findings were based on data from a convenience sample of female nursing students. The high level of health literacy may impact on weight perceptions and weight loss behaviours/intentions, and pressures on body image from society may have a different impact based on participants’ gender. Thus, generalizability of the study findings may be limited. Third, participants’ BMI was obtained by self-reported rather than objective measurements. People tend to overestimate their height and underestimate their weight in self-reports, resulting in a lower BMI than that found using objective measurements [33]. Nevertheless, a recent study conducted in 100 young adults (18–30 years) showed strong correlations between self-reported and objectively measured BMI (*r* = 0.94) [34]. Thus, self-reported height and weight are considered reliable when objective measurements are unavailable. Fourth, the two weight perception measures were presented in the same order for all participants, which might bring potential exposure effects. Finally, our study focused on the relationship between weight misperception and weight loss intentions, without examining the links to actual weight loss behaviours (such as fasting, taking diet pills or laxatives, inducing vomiting, or engaging in high-intensity physical activity). In the future, objective measurement of BMI should be used to obtain a clearer picture of weight perceptions measured by different methods. Longitudinal studies are warranted to explore the causal relationship between weight misperception and weight loss behaviours/intentions.

## 6. Conclusions

In conclusion, weight misperception is a common phenomenon among female college students, who tend to overestimate their weight status using verbal descriptions and underestimate their weight status using visual descriptions. Given that female students were more likely to accurately perceive their weight status with visual descriptions, especially in the underweight group, future large-scale studies can use visual descriptions to measure weight perception. Meanwhile, in-depth interviews are necessary to uncover reasons behind their perceptions, which may guide the development of interventional programs aimed at making positive lifestyle choices (e.g., avoiding excessive weight loss). Furthermore, weight misperception measured by verbal descriptions rather than visual descriptions strongly correlated with individuals’ actual weight loss intentions. These findings may help explain the discrepancies in results reported by previous studies and provide evidence for selecting appropriate weight perception measurements in clinical practice and future research.

## Figures and Tables

**Table 1:** The BMIs of each image in PFRS.

Image 1	Image 2	Image 3	Image 4	Image 5	Image 6	Image 7	Image 8	Image 9	Image 10
12.51	14.72	16.65	18.45	20.33	23.09	26.94	29.26	35.92	41.23

**Table 2:** Demographic characteristics of the participants (*n* = 600).

Variables	Characteristics	*n* (%)/Mean (SD)
Residence	Rural	218 (36.66)
	Urban	382 (63.67)
Living area	North	86 (14.33)
	Central	91 (15.17)
	South	423 (70.50)
Religion	Non-religious	552 (92.00)
	Religious	48 (8.00)
Nationality	Minority nationality	43 (7.17)
	Han nationality	557 (92.83)
Father’s level of education	Elementary school or below	84 (14.00)
	Junior high school	198 (33.00)
	Senior high school/technical secondary school	169 (28.17)
	Junior college or above	149 (24.83)
Mother’s level of education	Primary school or below	115 (19.17)
	Junior high school	206 (34.33)
	Senior high school/technical secondary school	167 (27.83)
	Junior college or above	112 (18.67)
Family monthly income	<5000 CNY	141 (23.50)
	5000–10000 CNY	242 (40.33)
	>10000 CNY	217 (36.17)
Drinking	Non-drinker	353 (58.83)
	Drinker or ex-drinker	247 (41.17)
Smoking	Non-smoker	576 (96.00)
	Smoker or ex-smoker	24 (4.00)
BMI status	Underweight	135 (22.50)
	Normal weight	394 (65.67)
	Overweight	60 (10.00)
	Obese	11 (1.83)
Ideal body shape	Underweight	538 (89.67)
	Normal weight	59 (9.83)
	Overweight/obese	3 (0.50)
Stress level	Never	100 (16.67)
	Mild	208 (34.67)
	Moderate	230 (38.33)
	Severe	62 (10.33)
Self-perceived weight level among peers	Slightly fat	222 (37.00)
	Moderate	279 (46.50)
	Slightly thin	99 (16.5)
Media influence on ideal body shape	High	135 (22.50)
	Moderate	352 (58.67)
	Infrequent	113 (18.83)
Weight loss intention	Always	112 (18.67)
	Often	128 (21.33)
	Sometimes	163 (27.17)
	Seldom	109 (18.17)
	Never	88 (14.67)
Peers’ attitudes		2.96 (0.66)
ATOP score		45.86 (9.46)

Notes: SD, standard deviation; ATOP, the attitudes toward obese persons; BMI, body mass index.

**Table 3:** Comparisons of participants’ BMI status with their perceived weight status (*n* = 600).

		BMI Status (*n*,%)				Kappa, κ
		UnderWeight	Normal Weight	Overweight	Obese	
**Weight perception measured by verbal descriptions**	Underweight	67 (49.63)	6 (1.52)	1 (1.67)	0 (0.00)	0.195
	Normal weight	56 (41.48)	161 (40.86)	1 (1.67)	0 (0.00)	
	Overweight	12 (8.89)	209 (53.05)	30 (50.00)	2 (18.18)	
	Obese	0 (0.00)	18 (4.57)	28 (46.67)	9 (81.82)	
**Weight perception measured by visual descriptions**	Underweight	119 (88.15)	163 (41.37)	0 (0.00)	0 (0.00)	0.304
	Normal weight	15 (11.11)	182 (46.19)	19 (31.67)	1 (9.09)	
	Overweight	1 (0.74)	33 (8.38)	19 (31.67)	0 (0.00)	
	Obese	0 (0.00)	16 (4.06)	22 (36.67)	10 (90.91)	

**Table 4:** Comparison of weight misperception measured by the two methods (*n* = 600).

		Weight Misperception Measured by Visual Descriptions			Total (*n*,%)
		Underestimated	Accurate	Overestimated	
**Weight misperception measured by verbal descriptions (*n*,%)**	Underestimated	6 (60.00)	4 (40.00)	0 (0.00)	10 (1.67)
	Accurate	110 (41.20)	142 (53.18)	15 (5.62)	267 (44.50)
	Overestimated	67 (20.74)	184 (56.97)	72 (22.9)	323 (53.83)
**Total (*n*,%)**		183(30.50)	330 (55.00)	87 (14.50)	600 (100.00)

**Table 5:** Ordinal logistic regression analyses of the association between weight misperception and weight loss intentions.

Independent Variables	β	S.E.	Wald χ2	*p*-Value	OR (95%CI)
**(1) Weight misperception measured by verbal descriptions (*n* = 590)**					
Overestimated	0.59	0.19	9.95	0.002	1.80 (1.25, 2.60)
Accurate (ref)	0				1.00
BMI status					
Overweight/obese	1.03	0.40	6.56	0.010	2.80 (1.27, 6.15)
Normal weight	0.93	0.27	12.39	<0.001	2.54 (1.51, 4.28)
Underweight (ref)	0				1.00
Self-perceived weight level among peers					
Slightly fat	1.59	0.38	17.83	<0.001	4.90 (2.34, 10.25)
Moderate	1.25	0.30	17.10	<0.001	3.50 (1.93, 6.35)
Slightly thin (ref)	0				1.00
Media influence on ideal body shape					
High	3.47	0.30	134.90	<0.001	32.17 (17.90, 57.80)
Moderate	1.52	0.23	43.88	<0.001	4.58 (2.92, 7.19)
Infrequent (ref)	0				1.00
Peers’ attitudes	0.73	0.13	31.12	<0.001	2.07 (1.60, 2.68)
ATOP score	−0.02	0.01	6.11	0.013	0.98 (0.96, 1.00)
**(2) Weight misperception measured by visual descriptions (*n* = 417)**					
Overestimated	0.14	0.25	0.29	0.587	1.14 (0.70, 1.86)
Accurate (ref)	0				1.00
Self-perceived weight level among peers					
Slightly fat	2.61	0.41	40.05	<0.001	13.59 (6.06, 30.45)
Moderate	1.84	0.34	29.85	<0.001	6.32 (3.26, 12.23)
Slightly thin (ref)	0				1.00
Media influence on ideal body shape					
High	3.30	0.36	86.10	<0.001	27.22 (13.54, 54.71)
Moderate	1.59	0.28	31.61	<0.001	4.92 (2.82, 8.57)
Infrequent (ref)	0				1.00
Peers’ attitudes	0.82	0.16	26.17	<0.001	2.27 (1.66, 3.11)
ATOP score	−0.02	0.01	5.04	0.025	0.98 (0.96, 1.00)

(1) Verbal descriptions: proportional odds assumption (χ2 = 45.86 [*p* = 0.677]); likelihood ratio test (χ2 = 415.24 [*p* < 0.001]); absence of multicollinearity between independent variables (the tolerance interval was [0.409, 0.893], and the interval of the variance inflation factor (VIF) was [1.119, 2.447]); (2) Visual descriptions: proportional odds assumption (χ2 = 58.07 [*p* = 0.547]); likelihood ratio test (χ2 = 305.68 [*p* < 0.001]); absence of multicollinearity between independent variables (the tolerance interval was [0.464, 0.908], and the interval of the VIF was [1.101, 2.154]); OR, odds ratio; CI, confidence interval; ref, reference.

## Data Availability

The data presented in this study are available on request from the corresponding author Daqiao Zhu. The data are not publicly available due to the privacy of the participants.
